# Arterio-Sclerosis and Blood Pressure

**Published:** 1918-08-31

**Authors:** 


### ARTERIO-SCLEROSIS AND BLOOD PRESSURE.

There are fashions in diagnosis as in other human enterprises, and some of them catch the public taste. One of the latest is arterio-sclerosis. The term has a sonorous quality and well fits the dignity of many a patient. In select pathological circles it has long had a fairly definite and demonstrable meaning. But it has now appeared in the clinical field, and it finds there a relatively large expansion. The physician may discover it, though he has no monopoly of the phrase, for the ophthalmologist puts in his claim with great confidence, and of course seeing is believing. Indeed, there are not a few departments of practice in which vague and mysterious symptoms are held to find their interpretation in this commanding diagnosis. Speaking generally, it is not infrequent to find that the diagnosis rests upon observations made by the sphygmometer, and the general conviction seems to be that when this instrument registers what is regarded as an unduly high reading, such reading is an announcement of the hardening by degenerative changes of the tissues of the arterial walls. When, in other words, the measurement named blood pressure is above what is fixed as normal for the age of the patient, this is both the sign and the consequence of arterio-sclerosis. There are, however, certain circumstances which give pause to this conclusion. The most impressive is the fact that in cases not a few, though arterial degeneration is visible and palpable, there is no abnormal increase of the sphygmometer reading. On the other hand, it is not less true that this reading may be high, although the arteries, so far as they are accessible to examination, appear to be of normal texture. Hence it is surely manifest that the belief that arterio-sclerosis and so-called high blood pressure are parallel and relative events is not well founded. Either may exist without the other, and to read from the sphygmometer either to the absence or to the presence of arterial degeneration is not justified by the facts.

The truth is that the sphygmometer is an apparatus so little in need of technical skill, and the inference that hardening of the arterial walls must needs be accompanied by an increase of pressure is so plausible a one, that it is easy to slip into a confident conclusion. Hence arises the wide extension of the diagnosis of arterio-sclerosis and its prevalence among the public. That degenerative changes may occur, and often do occur, in the arterial walls no one for a moment disputes. But what is necessary to recognise is that the sphygmometer reading is not the measure of them. Nor is this a mere matter of academic doctrine. On the contrary, the conditions which give rise to high sphygmometer records may be not the consequence but the cause of arterial degenerative change, and from this there follows necessarily a practical therapeutic position. For such changes, when they have once occurred, can hardly hope for removal. But if the high readings which are often interpreted as arterio-sclerosis mean only the existence of stresses likely to lead to this condition, treatment may find a large opportunity in the removal of these stresses. What is to be desired is that arterio-sclerosis shall not be accepted as a diagnosis too readily; that when it exists the circumstances responsible for it shall be carefully considered as the condition has not in every instance one and the same origin; and, still more, that high sphygmometer readings shall, in the absence of evidence of renal and arterial change, be interpreted as a claim for the institution of measures likely to prevent the development of vascular degeneration.

